# The Impact of a Standardized Pre-visit Laboratory Testing Panel in the Internal Medicine Outpatient Clinic: a Controlled “On-Off” Trial

**DOI:** 10.1007/s11606-020-06453-2

**Published:** 2021-01-22

**Authors:** B. E. L. Vrijsen, M. J. ten Berg, C. A. Naaktgeboren, J. Y. Vis, H. M. Dijstelbloem, J. Westerink, D. Dekker, I. E. Hoefer, S. Haitjema, C. A. R. Hulsbergen-Veelken, W. W. van Solinge, H. A. H. Kaasjager

**Affiliations:** 1 Department of Internal Medicine, Division Internal Medicine and Dermatology, University Medical Center Utrecht, Utrecht University, F02.216, PO Box 85500, 3508 GA Utrecht, the Netherlands; 2 Central Diagnostic Laboratory, Division Laboratories, Pharmacy and Biomedical Genetics, University Medical Center Utrecht, Utrecht University, Utrecht, the Netherlands; 3 Julius Center for Health Sciences and Primary Care, University Medical Center Utrecht, Utrecht University, Utrecht, the Netherlands; 4 Department of Clinical Chemistry, Erasmus MC, University Medical Center Rotterdam, Rotterdam, the Netherlands

**Keywords:** diagnosis, clinical chemistry tests, ambulatory care

## Abstract

**Background:**

In several settings, a shorter time to diagnosis has been shown to lead to improved clinical outcomes. The implementation of a rapid laboratory testing allows for a pre-visit testing in the outpatient clinic, meaning that test results are available during the first outpatient visit.

**Objective:**

To determine whether the pre-visit laboratory testing leads to a shorter time to diagnosis in the general internal medicine outpatient clinic.

**Design:**

An “on-off” trial, allocating subjects to one of two treatment arms in consecutive alternating blocks.

**Participants:**

All new referrals to the internal medicine outpatient clinic of a university hospital were included, excluding second opinions. A total of 595 patients were eligible; one person declined to participate, leaving data from 594 patients for analysis.

**Intervention:**

In the intervention group, patients had a standardized pre-visit laboratory testing before the first visit.

**Main Measures:**

The primary outcome was the time to diagnosis. Secondary outcomes were the correctness of the preliminary diagnosis on the first day, health care utilization, and patient and physician satisfaction.

**Key Results:**

There was no difference in time to diagnosis between the two groups (median 35 days vs 35 days; hazard ratio 1.03 [0.87–1.22]; *p* = .71). The pre-visit testing group had higher proportions of both correct preliminary diagnoses on day 1 (24% vs 14%; *p* = .003) and diagnostic workups being completed on day 1 (10% vs 3%; *p* < .001). The intervention group had more laboratory tests done (50.0 [interquartile range (IQR) 39.0–69.0] vs 43.0 [IQR 31.0–68.5]; *p* < .001). Otherwise, there were no differences between the groups.

**Conclusions:**

Pre-visit testing did not lead to a shorter overall time to diagnosis. However, a greater proportion of patients had a correct diagnosis on the first day. Further studies should focus on customizing pre-visit laboratory panels, to improve their efficacy.

**Trial Registration:**

NL5009

**Supplementary Information:**

The online version contains supplementary material available at 10.1007/s11606-020-06453-2.

## INTRODUCTION

A shorter time to diagnosis has been shown to lead to improved clinical outcomes in some, but not all health care settings.^1–4^ Given that the majority of medical decisions rely on a laboratory testing,^5^ speeding up access to laboratory results may lead to earlier diagnoses. For instance, faster turnaround times of microbiology tests have been shown to lead to faster initiation of adequate antibiotic therapy and shorter inpatient length of stay.^6,7^

One way to speed up the diagnostic process is through a pre-visit laboratory testing, in which patients have the laboratory testing done directly prior to their doctor’s appointment, and the tests are performed with a short turnaround time at a routine clinical laboratory. By necessity, the pre-visit testing makes use of standardized laboratory test panels, as the patient has not been examined yet so the information from the history and physical examination is not yet available to guide the selection of laboratory tests. Standardized laboratory panels invariably include unnecessary tests, which potentially lead to downstream overutilization.^8^

In the Netherlands, the pre-visit testing is not the currently standard practice in the outpatient clinic, due to the often long turnaround times in the laboratory.

In 2014, the Central Diagnostic Laboratory of the University Medical Centre Utrecht (UMC Utrecht) introduced a rapid testing for a broad panel of routine laboratory tests for clinical chemistry, hematology, coagulation, and endocrinology. This service guarantees that the results of these tests are available within 60 min after the sample arrives at the laboratory. This rapid testing enables the implementation of the pre-visit testing in the outpatient clinic, making the test results available to the treating physician at the time of the visit, as opposed to usual care, in which the treating physician orders laboratory tests during the visit and the test results are only available afterwards, so the patient has to return for another visit.

Consequently, this “on-off” trial was set up to evaluate whether the pre-visit laboratory testing benefits the patient and physician alike, shortening the time to diagnosis in newly referred outpatients, and to evaluate the downstream consequences of potential overutilization.

## METHODS

### Trial Design

This is a single-center controlled “on-off” trial, in which patients were alternately allocated to the intervention group or the usual care group in 3-month blocks.^9^ Subjects were not randomized individually, because it was not feasible to incorporate the two different testing strategies in the laboratory order management system simultaneously.

In the intervention group, patients had laboratory tests performed directly prior to their first visit to the outpatient clinic, and in the usual care group, laboratory tests were done afterwards.

### Patient Selection

All adult patients newly referred to the general internal medicine outpatient clinic of the University Medical Center Utrecht (UMC Utrecht), a large university hospital in Utrecht, the Netherlands, were eligible for inclusion. Referrals for second opinions were excluded because these patients often already have a diagnosis.

In the Netherlands, patients require a referral from their primary care physician before they visit an outpatient clinic. This referral consists of a letter with the reason of referral, sometimes accompanied by laboratory results, but not with the entire patient record due to privacy regulations. The general internal medicine outpatient clinic of the UMC Utrecht receives the primary care physicians’ referrals electronically (and by regular mail). New referrals are triaged by an attending physician and are then randomly assigned to either a resident or an attending physician for an outpatient consultation. The residents are required to confer all consultations with their supervising attendings.

Based on an estimated sample size of 460 participants, four 3-month blocks were initially planned from April 2015 to April 2017. However, because inclusion went slower than previously anticipated, the inclusion period was extended to August 2017 by adding two 2-month blocks.

### Intervention

In the intervention group, patients were asked to have their blood drawn 1 h before their scheduled appointment for their first visit, so that the treating physician had access to the test results during the visit. All patients in the intervention group received the same standard laboratory panel, regardless of the referral reason. This panel had been established before the start of the trial by a panel of experienced internists and clinical chemists and comprised the following tests: hemoglobin, cell counts/differential, sodium, potassium, calcium, urea, creatinine, alkaline phosphatase, gamma-glutamyl transferase (GGT), glucose, aspartate transaminase (AST), alanine transaminase (ALT), lactate dehydrogenase (LDH), albumin, C-reactive protein (CRP), thyroid-stimulating hormone (TSH), erythrocyte sedimentation rate (ESR), and a urine strip screening.

In the usual care group, laboratory tests were ordered at the discretion of the treating physician during the first visit and test results were only available afterwards.

In both groups, all other aspects of medical care, such as imaging tests and the planning of follow-up visits, were at the discretion of the treating physician.

### Outcomes and Measures

#### Baseline Characteristics

For all patients, age at referral and gender were retrieved from the Utrecht Patient Oriented Database (UPOD), an infrastructure of relational databases comprising data on patient characteristics, hospital discharge diagnoses, medical procedures, medication orders, and laboratory tests for all patients treated at UMC Utrecht. UPOD data acquisition and management is in accordance with current regulations concerning privacy and ethics. The structure and content of UPOD have been described in more detail elsewhere.^10^ Furthermore, whether the physician performing the first consultation was a resident or an attending was obtained from the patient charts. Referral reasons and, if available, the results of the laboratory testing performed by the referring physician were taken from the referral letter. Referral reasons were grouped into the following categories: anemia, fatigue, weight loss, gastro-intestinal complaints, abnormal laboratory test result(s) (other than anemia), lymphadenopathy/suspected malignancy, and other. These categories were non-exclusive as some patients had more than one referral reason.

#### Primary Outcome

The primary outcome was the time to diagnosis, defined as the number of days between the first visit to the outpatient clinic and the final diagnosis being made. The date of final diagnosis was defined as the date the patient was informed of the diagnosis and after which no further tests or examinations were performed to confirm or refute this diagnosis. If a patient had more than one diagnosis, the date of the last diagnosis was used.

An expert panel of internal medicine physicians assessed the date of the final diagnosis. Assessment was planned 2 years after the patient’s initial visit. Each case was individually assessed by two panelists, and a third panelist was consulted when there was a disagreement. If two out of three panelists agreed on a date, this date was chosen. Cases where none of the panelists agreed on a date were resolved by consensus through discussion between the panelists. Inter-rater agreement was evaluated using the one-way random effects intra-class coefficient.^11^

#### Secondary Outcomes

Secondary outcomes were the correctness of the preliminary diagnosis at the first visit to the outpatient clinic, utilization of health care resources during the diagnostic process, and patient and physician satisfaction.

### Correctness of the Preliminary Diagnosis

The correctness of the preliminary diagnosis on the first day was assessed using two parameters: firstly, the proportion of patients in whom the treating physician’s preliminary diagnosis at the first visit agreed with the final diagnosis, and secondly, the proportion of patients in whom the diagnostic process was completed on the first day.

### Utilization of Health Care Resources

Health care utilization was measured by the number of medical procedures during the diagnostic process, including the pre-visit laboratory tests. Specifically, the number of outpatient clinic visits, clinical admissions, laboratory tests, venipunctures, imaging, and endoscopies were collected from the UPOD database. Furthermore, the physicians were asked to report the duration of the first consultation at the outpatient clinic, in minutes.

### Patient and Physician Satisfaction

Patient satisfaction was assessed through a questionnaire, which was handed out to patients at their first visit to the outpatient clinic. Patients were asked about their preferences regarding laboratory testing strategies, as well as their satisfaction with their first visit to the outpatient clinic by an overall grade (1–10) and by using a modified Patient Satisfaction Questionnaire Short-Form (PSQ-18)^12^ that excluded questions on financial consequences and accessibility of health care, as these items were not thought to be relevant in this setting.

Physicians were asked three yes-or-no questions regarding satisfaction with testing strategies after the first visit: whether they had a good overview of the patient’s problem, whether they were able to help the patient efficiently, and whether the diagnosis was already in sight.

### Statistical Analyses

A sample of 200 patients per treatment arm was required to detect a 7-day difference in the mean time to final diagnosis with a power of 80% and an alpha of .05, assuming a standard deviation of 25 days. To compensate for the non-normality of the data, the required sample size was increased by 15%, yielding a required total sample size of 230 patients in each group.^13^ All analyses were done according to the intention-to-treat principle.

Survival analysis was used to evaluate the primary outcome (time to diagnosis) to account for censoring due to loss to follow-up. A hazard ratio was calculated using Cox proportional hazards analysis. A Kaplan-Meier plot as well as medians and interquartile ranges for the time to diagnosis was also reported to aid with interpretation of the data.

Subgroup analyses of the primary outcome were performed on gender, referral reason, whether the referral letter contained the results of the laboratory testing by the referring physician, and whether the physician who treated the patient at the first visit to the outpatient clinic was a resident or attending physician.

The rates of correct preliminary diagnoses on the first day and diagnostic processes being completed on the first day were tested with Pearson’s *χ*^2^ test. The number of medical procedures in the diagnostic process was tested using negative binomial regression. Patient preferences were tested using the Wilcoxon-Mann-Whitney test and patient satisfaction was tested using Student’s *t* test. Physician preferences were tested using Pearson’s *χ*^2^ test. The duration of the first visit was tested using Student’s *t* test.

All statistical analyses were performed in R version 3.5.1.^14^

### Ethical Considerations

Because both laboratory testing strategies we investigated were already used in clinical practice and no other burden was imposed on patients other than the questionnaire, the institutional review board waived the requirement for informed consent. Patients had the opportunity to opt out of the study. The study was registered in the Netherlands Trial Register, number NL5009.

## RESULTS

### Baseline Characteristics

**Figure 1:**
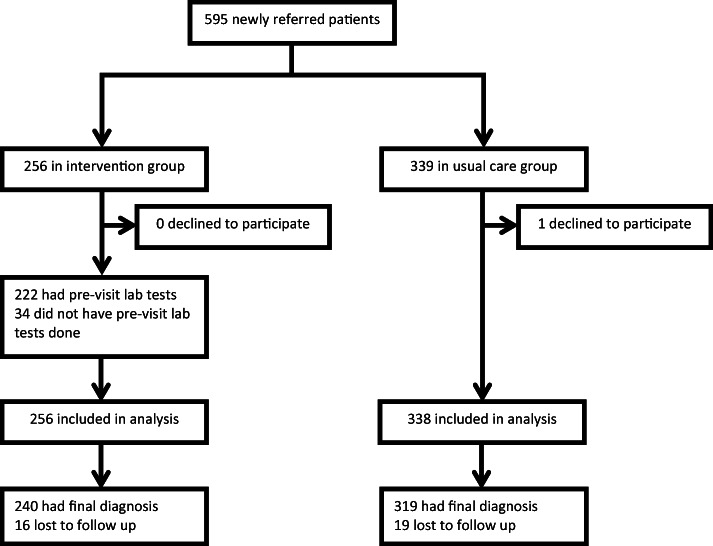
**Inclusion flowchart.**

In total, 595 patients were eligible for inclusion. One patient in the usual care group declined to participate, which left 594 patients for inclusion: 256 in the intervention group and 338 in the usual care group. Thirty-four patients (13%) in the intervention group erroneously did not have pre-visit laboratory testing done. All 594 patients were included in the analyses. A flowchart of the inclusions is provided in Figure 1. Baseline characteristics are presented in Table 1. The mean time between the initial visit and the expert panel’s assessment was 713 days (95% CI 701–726 days). Loss to follow-up was 6% after a median follow-up time of 138 days (43–270).

**Table 1:** Baseline Characteristics

	Pre-visit testing (*n* = 256)	Usual care (*n* = 338)
Female gender	145 (57%)	207 (62%)
Age	53.9 (51.7–56.1)	51.7 (49.8–53.7)
Referral reason (grouped)*		
Abnormal lab test	55 (21%)	45 (13%)
Anemia	28 (11%)	43 (13%)
Fatigue	64 (25%)	83 (25%)
Gastro-intestinal complaints	24 (9%)	41 (12%)
Lymphadenopathy/suspected malignancy	15 (6%)	17 (5%)
Weight loss	26 (10%)	42 (12%)
Other	65 (25%)	88 (26%)
Availability of pre-referral laboratory test results	121 (47%)	174 (51%)
Seen by attending physician	27 (11%)	50 (15%)

*Categories are non-exclusive

### Time to Diagnosis

**Figure 2:**
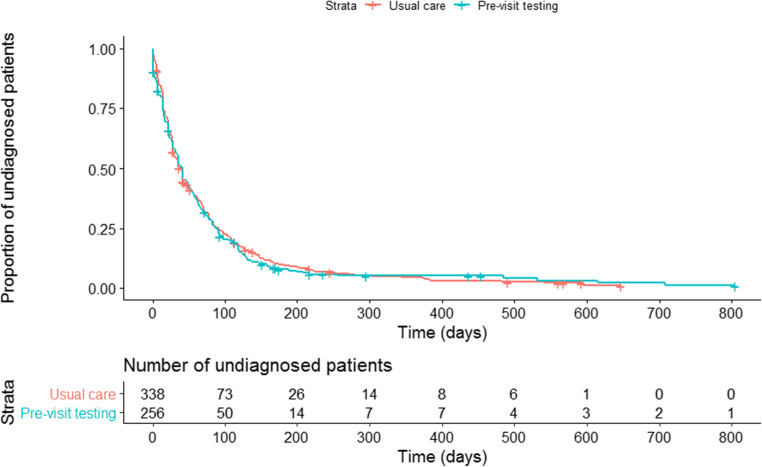
**The Kaplan-Meier plot of time to diagnosis in days. Hazard ratio 1.03 (0.87–1.22);** ***p*** **= .71.**

There was no difference in time to diagnosis (in days) between the two groups (Fig. 2; hazard ratio 1.03 [0.87–1.22]; *p* = .71). A definitive diagnosis was made in 94% of patients. For these patients, median time to diagnosis was 35.0 days (interquartile range 14.0–77.3) in the intervention group and 35.0 days (IQR 14.0–83.0) in the control group. A list of the final diagnoses is provided in Supplementary Table 1.

When establishing the time to diagnosis, the two experts agreed in 408 cases (69%). When a third reviewer was necessary to resolve discrepancies, there was an agreement between two of the three experts in 551 cases (77%); the remaining 43 cases (23%) were resolved through a discussion. The one-way random effects intra-class coefficient was 0.63, indicating a moderate inter-rater agreement.

There were no differences in time to diagnosis between the intervention and control groups for subgroups based on gender, referral reason, the availability of the results of the pre-referral laboratory testing by the referring physician, or whether the initial consultation was done by a resident or an attending physician (Supplementary Table 2).

### Diagnosis After First Visit

The rate of agreement between the treating physician’s preliminary diagnosis on the first day and the definitive diagnosis at the end of the diagnostic process was 24% in the intervention group versus 14% in the control group (*p* = .003). The proportion of patients in whom the diagnostic process was completed on the first day was also significantly higher in the intervention group (10% vs 3%; *p* < .001).

### Health Care Utilization

Patients in the intervention group had more laboratory tests done, both on the day of the first visit (median 22.0 [21.0–26.0] vs 20.0 [10.0–26.0]; *p* < .001) and during the entire diagnostic process (median 50.0 [39.0–69.0] vs 43.0 [31.0–68.5]; *p* = .001). There were no other differences in the number of medical procedures during the time to diagnosis. Notably, the total number of visits to the outpatient clinic was similar in both groups (2.0 [1.8–4.0] vs 2.0 [2.0–4.0]; *p* = .63). The first visit did last slightly longer in the intervention group (48.3 [46.6–49.9] vs 45.0 [42.3–46.7] min; *p* = .006). All data on health care utilization are presented in Table 2.

**Table 2:** Health Care Utilization During the Diagnostic Process

	Pre-visit testing (*n* = 256)	Usual care (*n* = 338)	
	Medians + interquartile ranges	Medians + interquartile ranges	
Visits to out-patient clinic			
- Any	2.0 (1.8–4.0)	2.0 (2.0–4.0)	*p* = .63*
- Internal medicine	2.0 (1.0–3.0)	2.0 (1.0–3.0)	*p* = .96*
Teleconsultations	1.0 (0.0–3.0)	1.0 (0.0–2.0)	*p* = .70*
Number of clinical admissions	0.0 (0.0–1.0)	0.0 (0.0–1.0)	*p* = .37*
Clinical admission days	0.0 (0.0–1.0)	0.0 (0.0–1.0)	*p* = .42*
Laboratory tests (total)	50.0 (39.0–69.0)	43.0 (31.0–68.5)	*p* = .001*
Laboratory tests (first day)	22.0 (21.0–26.0)	20.0 (10.0–26.0)	*p* < .001*
Laboratory test orders	3.5 (2.0–5.3)	3.0 (2.0–5.0)	*p* = .11*
Imaging tests	1.0 (0.0–3.0)	1.0 (0.0–2.0)	*p* = .46*
Number of patients with imaging tests	*n* (%)	*n* (%)	
Any imaging	180 (70%)	222 (66%)	*p* = .27^†^
MRI	20 (8%)	21 (6%)	*p* = .55^†^
CT	62 (24%)	68 (20%)	*p* = .27^†^
Ultrasound	84 (33%)	108 (33%)	*p* = .89^†^
Nuclear	19 (7%)	18 (5%)	*p* = .38^†^
X-ray	111 (43%)	144 (43%)	*p* = .92^†^
Endoscopy	34 (13%)	57 (17%)	*p* = .28^†^

*MRI*, magnetic resonance imaging; *CT*, computed tomography

*Negative binomial regression

^†^Pearson’s *χ*^2^ test

Additionally, a post hoc analysis to assess the adequacy of the standard laboratory panel showed that in 66% of subjects in the intervention group, additional laboratory tests were ordered on the first day (Supplementary Table 3).

### Satisfaction

There was no difference in physician and patient satisfaction between the two groups (Tables 3 and 4). However, there were some differences in patients’ preferences (Supplementary Figure 1): patients who had had pre-visit laboratory tests done were more likely to want to learn the diagnosis on the same day (93% vs 91%; *p* < .001), and less likely to want to see the doctor before having laboratory tests done (17% vs 43%; *p* < .001). However, they were more likely to object to more laboratory tests being done than necessary (16% vs 8%; *p* = .01).

**Table 3:** Physician Satisfaction

	Pre-visit testing (*n* = 191) (% of respondents answering affirmatively)	Usual care (*n* = 161) (% of respondents answering affirmatively)	
Good overview of the problem?	98%	98%	*p* > .99
Able to help the patient efficiently?	65%	71%	*p* = .28
Diagnosis already in sight?	57%	50%	*p* = .24

Differences between the groups were tested using Pearson’s *χ*^2^ test

**Table 4:** Patient Satisfaction

Respondents, *n* (%)	Pre-visit testing (*n* = 125 [49%]) (means + 95% confidence interval)	Usual care (*n* = 131 [39%]) (means + 95% confidence interval)	
Overall grade	8.0 (7.8–8.2)	8.1 (7.9–8.3)	*p* = .54
Modified PSQ-18 questionnaire			
General satisfaction	3.85 (3.71–3.98)	3.81 (3.66–3.96)	*p* = .72
Technical quality	3.86 (3.75–3.97)	3.73 (3.62–3.84)	*p* = .11
Interpersonal manner	4.35 (4.02–4.26)	4.20 (4.07–4.33)	*p* = .07
Communication	4.14 (4.02–4.26)	4.00 (3.87–4.12)	*p* = .10
Time spent with doctor	4.02 (3.90–4.15)	3.84 (3.70–3.98)	*p* = .05

Differences between the groups were tested using Student’s *t* test. Items of the PSQ-18 are scored on a 1–5 scale, with high scores reflecting greater satisfaction with medical care

## DISCUSSION

In our single-center on-off study, performing a standardized laboratory test panel prior to the first visit to the outpatient clinic did not result in a shorter overall time to diagnosis. However, it did increase the chance of obtaining a final diagnosis during the first visit. The number needed to test in order to finish the diagnostic process on the first day was 15. In our questionnaire, the vast majority of patients preferred receiving their diagnosis on the first day, and at the same time did not object to having more laboratory tests done than necessary.

Ordering standardized laboratory panels is in sharp contrast with advice from several guidelines, including Choosing Wisely recommendations, because of potential overutilization.^15^ Overutilization leads to increased costs, as well as potentially more false positive test results.^16^

In this study, patients in the intervention group on average had 7 more laboratory tests performed during the diagnostic process. No other differences in health care utilization were found, which implies that the excess laboratory tests did not lead to significant downstream overutilization.

To the best of our knowledge, this is the first time that the effect of the pre-visit laboratory testing on the diagnostic process in the outpatient setting has been studied.

Several possible explanations for the study’s negative result can be proposed. First of all, laboratory testing may not be as important for establishing a diagnosis as previously hypothesized. This might be especially true in the setting of a tertiary hospital, which typically has a more complex case mix that requires a more extensive diagnostic workup. This might also explain why the pre-visit testing would have an effect on the number of correct diagnoses on the first day, as in these cases typically no additional diagnostic tests were performed. It might also be argued that the laboratory testing already performed by the referring physician negated the effect of the pre-visit testing, although in that case one would have expected to see an effect in the subgroup without pre-referral testing.

Alternatively, given that additional tests were ordered after the first visit in 66% of cases in the pre-visit arm, it might also be argued that the pre-visit panel proved inadequate in those patients and that a different or more extensive test panel would have made a difference. However, extending the pre-visit panel would lead to greater costs and possibly more downstream overutilization. Tailoring the pre-visit panel to individual patients based on their referral reason might be a more promising alternative.

One of the strengths of this study is that all subjects were comprehensively analyzed through a chart review by an expert panel. As a result, loss to follow-up was limited at 6%. Furthermore, the study population was relatively unselected, as all newly referred patients were included, which increases the study’s external validity.

This study has several limitations. Firstly, the on-off trial design may have compromised the comparability of the two groups.

Secondly, this was an open-label study. The subjects and their treating physicians were aware of their allocation. The expert panelists who determined the time to diagnosis were not actively informed about the allocation of the subjects they evaluated, but in many cases, it could be inferred from the treating physician’s chart notes they reviewed.

Thirdly, the response rate of the patient survey was quite low at 49% of patients in the intervention group and 39% in the control group, which could limit the generalizability of the questionnaire’s results.

Fourthly, the physician survey comprised only three questions, because we presumed that physicians would not be willing to fill out longer surveys. This may limit the survey’s applicability.

Finally, even though we found no differences in health care utilization apart for a modest increase in the number of laboratory tests in the intervention group, not all potentially negative effects of test overutilization, such as anxiety due to false positive test results, were monitored for in this study.

In conclusion, the standardized pre-visit laboratory testing did not lead to a shorter time to diagnosis but increased the chance of obtaining the correct diagnosis during the first visit. Further studies should focus on adaptations and differentiations to the standard pre-visit laboratory panel.

## Supplementary Information

ESM 1 (DOCX 267 kb)
