# Supplementary material for: The Impact of a Standardized Pre-visit Laboratory Testing Panel in the Internal Medicine Outpatient Clinic: a Controlled “On-Off” Trial
Source: J Gen Intern Med. 2021 Jan 22;36(7):1914–20. doi: 10.1007/s11606-020-06453-2 (PMC8298644; doi:10.1007/s11606-020-06453-2)
Supplement: Supplementary file 1 — (DOCX 267 kb) [file 11606_2020_6453_MOESM1_ESM.docx]

## Supplementary

Supplementary Table 1: List of final diagnoses

Supplementary Table 2: Time to diagnosis in subgroups

Supplementary Table 3: Laboratory tests performed in addition to the standard pre-visit panel

Supplementary Figure 1: Patient preferences

### Supplementary Table 1: List of final diagnoses

Abbreviations: ACNES: abdominal cutaneous nerve entrapment syndrome; BPPV: benign paroxysmal postural vertigo; COPD: chronic obstructive pulmonary disease; MGUS: monoclonal gammopathy of unknown significance; PFAPA: periodic fever, aphthosis, pharyngitis, and adenitis; SADNI: selective antibody deficiency with normal immunoglobulins; SIADH: syndrome of inappropriate antidiuretic hormone secretion

45 patients had more than one diagnosis.

### Supplementary Table 2: Time to diagnosis in subgroups

|  | Number | Median time to diagnosis  (+ interquartile range)* | | Hazard ratio† |
| --- | --- | --- | --- | --- |
|  |  | Pre-visit testing | Usual care |  |
| **All** | 594 | 35.0 (14.0-77.3) | 35.0 (14.0-83.0) | 1.03 (0.87-1.22); p=.71 |
| **Gender** |  |  |  |  |
| Female |  | 35.0 (14.0-83.3) | 35.0 (14.0-83.5) | 0.95 (0.77-1.19); p=.68 |
| Male |  | 36.5 (14.0-68.0) | 35.0 (14.0-81.5) | 1.16 (0.89-1.51); p=.26 |
| **Referral reason**‡ |  |  |  |  |
| Abnormal lab test | 100 | 28.5 (1.0-57.0) | 21.0 (7.0-78.8) | 1.40 (0.92-2.13); p=.12 |
| Anemia | 71 | 53.0 (20.0-105.0) | 55.0 (23.5-116.0) | 0.85 (0.50-1.43); p=.54 |
| Fatigue | 147 | 42.0 (19.5-81.5) | 42.0 (20.3-73.0) | 0.93 (0.67-1.31); p=.69 |
| Gastro-intestinal complaints | 65 | 43.0 (15.5-67.5) | 49.0 (26.3-90.8) | 1.38 (0.82-2.34); p=.23 |
| Lymphadenopathy / suspected malignancy | 32 | 22.0 (14.0-66.5) | 27.5 (8.8-46.3) | 0.83 (0.40-1.72); p=.61 |
| Weight loss | 68 | 41.0 (16.5-84.5) | 35.0 (16.0-112.0) | 1.67 (0.98-2.84); p=.06 |
| Other | 153 | 34.5 (14.8-74.3) | 28.0 (14.0-77.3) | 0.77 (0.55-1.08); p=.13 |
| **Availability of the results of pre-referral laboratory testing by referring physician**  Yes  No | 299  295 | 42.0 (14.0-88.0)  35.0 (14.0-82.5) | 32.5 (14.0-77.0)  40.5 (14.0-101.5) | 0.89 (0.70-1.13); p=.32  1.19 (0.94-1.51); p=.15 |
| **Patient seen by** |  |  |  |  |
| Resident | 517 | 35.0 (14.0-73.0) | 34.0 (14.0-79.0) | 1.06 (0.65-1.75); p=.81 |
| Attending physician | 77 | 63.0 (33.5-95.0) | 52.5 (19.3-94.8) | 1.00 (0.84-1.20); p=.97 |

* for completed cases

† p-values from Cox regression

### Supplementary Table 3: Laboratory tests performed on the first day in addition to the standard pre-visit panel

| **Additional test** | **number of times performed** |
| --- | --- |
| Ferritin | 70 |
| Vitamin B12 | 46 |
| Folic acid | 42 |
| Free thyroxine (FT4) | 30 |
| Creatine kinase | 28 |
| Phosphate | 22 |
| Bilirubin (total) | 19 |
| M-protein | 19 |
| Sodium | 17 |
| Total protein | 17 |
| Iron | 15 |
| 25-hydroxyvitamin D | 14 |
| Osmolality | 14 |
| Creatinine (urine) | 13 |
| Osmolality (urine) | 13 |
| Reticulocytes | 13 |
| Magnesium | 12 |
| Transferrin | 10 |
| Transferrin saturation | 10 |
| Glycated hemoglobin (HbA1c) | 9 |
| Anti-endomysium | 8 |
| Anti-nuclear antibodies | 8 |
| Anti-Tissue transglutaminase (TTG) | 8 |
| Cholesterol | 7 |
| High density lipoprotein (HDL) cholesterol | 7 |
| Low density lipoprotein (LDL) Cholesterol | 7 |
| Triglycerides | 7 |
| Bicarbonate | 6 |
| Haptoglobin | 6 |
| Immunoglobulin A | 6 |
| Prothrombin time | 6 |
| Anti-ds-DNA | 5 |
| Cortisol | 5 |
| Immunoglobin G | 5 |
| Lipase | 5 |
| Potassium | 5 |
| Urea | 5 |
| Activated partial thromboplastin time (APTT) | 4 |
| Albumin (urine) | 4 |
| Anti-smooth muscle antibodies | 4 |
| Erythropoietin | 4 |
| International normalized ratio (INR) | 4 |
| Rheumatoid factor | 4 |
| Amylase | 3 |
| Angiotensin-converting-enzyme (ACE) | 3 |
| Anti-mitochondrial antibodies | 3 |
| Citrullin | 3 |
| Immunoglobulin M | 3 |
| Lactate | 3 |
| Parathyroid hormone | 3 |
| Red cell distribution width | 3 |
| Reticulocytes mean corpuscular hemoglobin concentration | 3 |
| Thyroid-stimulating immunoglobulin | 3 |
| Adrenocorticotropic hormone (ACTH) | 2 |
| Anti-thyroid peroxidase (TPO) antibodies | 2 |
| B-type natriuretic peptide (BNP) | 2 |
| Complement C3 | 2 |
| Cytoplasmic anti-neutrophil cytoplasmic antibodies (cANCA) | 2 |
| Dysmorphic erythrocytes (urine) | 2 |
| Free light chains | 2 |
| Insulin | 2 |
| Intrinsic factor | 2 |
| Perinuclear anti-neutrophil cytoplasmic antibodies (pANCA) | 2 |
| Prostate-specific antigen (PSA) | 2 |
| Testosterone | 2 |
| Thrombocytes (citrate) | 2 |
| Thrombocytes (heparin) | 2 |
| Type and screen | 2 |
| Uric acid | 2 |
| Vitamin B6 | 2 |

## Total group size is 222 subjects. Only tests ordered more than once are included.

### Supplementary Figure 1: Patient preferences


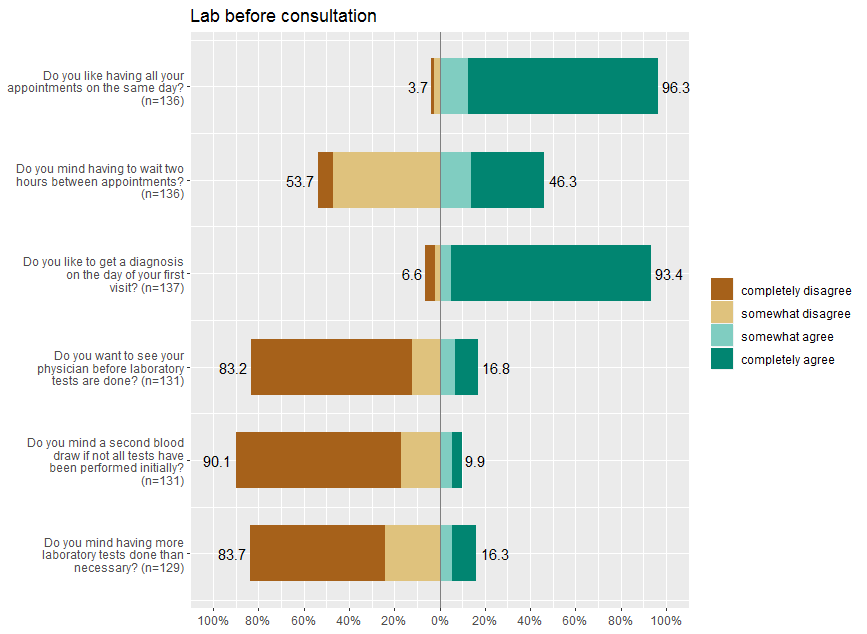

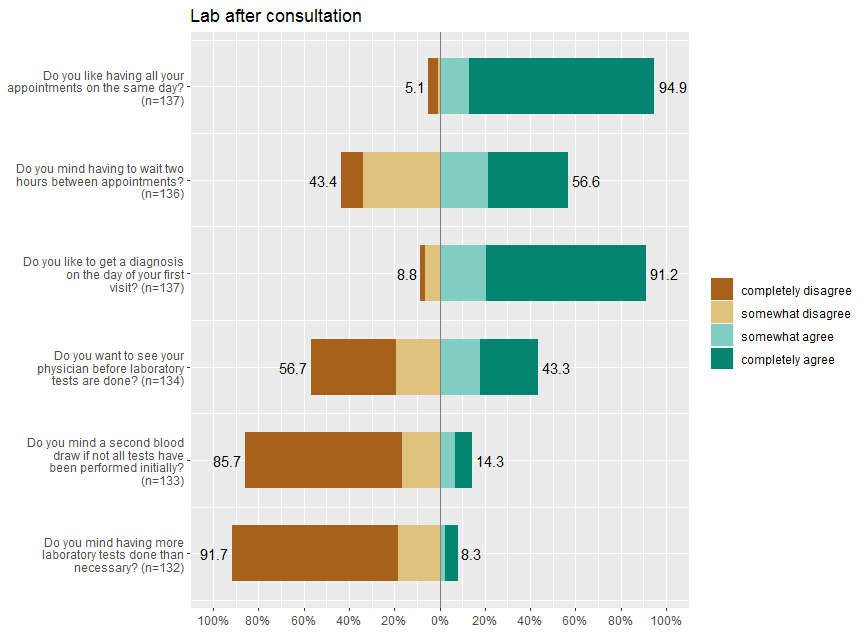


p=.62

p=.36

p<.001

p<.001

p=.38

p=.01

(Comparisons between groups were tested by Mann-Whitney-Wilcoxon test)


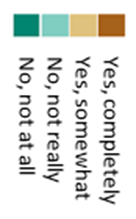


Pre-visit testing

Usual care
